# Resection of solitary abdominal wall metastasis of ascending colon cancer along the ventriculoperitoneal shunt: A case report

**DOI:** 10.1016/j.ijscr.2021.105869

**Published:** 2021-04-07

**Authors:** Masahiro Kataoka, Hiroka Kondo, Yasumitsu Hirano

**Affiliations:** Saitama Medical University International Medical Center, Department of Gastroenterological Surgery, 1397-1 Yamane, Hidaka, Saitama, Japan

**Keywords:** VP, ventriculoperitoneal, UICC, the Union for International Cancer Control, CEA, carcinoembryonic antigen, CT, computed tomography, FDG-PET/CT, ^18^F-fluorodesoxyglucose positron-emission tomography/computed tomography, H&E, hematoxylin and eosin, Ventriculoperitoneal shunt, Abdominal wall metastasis, Ascending colon cancer

## Abstract

**Introduction:**

Ventriculoperitoneal (VP) shunt is often placed as a treatment for hydrocephalus. Additionally, it is also not uncommon to perform laparoscopic surgery for colon cancer with a VP shunt in place. It is very rare for colorectal cancer to metastasize to an implanted VP shunt. We report a case of VP shunt-related metastasis of the ascending colon that was successfully resected.

**Presentation of case:**

A 79-year-old man who had a VP shunt for hydrocephalus two years earlier underwent laparoscopic right colectomy for ascending colon cancer. Six months after the colectomy, imaging examinations showed mass formation in the subcutaneous tissue of the abdominal wall along the VP shunt. Because of the possible metastasis of colorectal cancer and the fact that it was a solitary lesion, a tumor resection with replacement of the VP shunt was performed. Histopathological examination revealed that the mass was a metastasis of colon cancer.

**Discussion:**

This case involves the metastasis of colorectal cancer in the subcutaneous tissue of the abdominal wall after laparoscopic surgery, and since the tumor had reached the serosa, the possibility of metastasis by pneumoperitoneum was considered. Careful intraoperative manipulation is considered to be important for prevention.

**Conclusion:**

Metastasis of colorectal cancer to the VP shunt is extremely rare, but possible. In such cases, intraoperative prevention and careful postoperative follow-up are required.

## Highlights

• Colorectal cancer could metastasize to the VP shunt, although it is rare.
• Careful intraoperative manipulation would prevent metastasis to the VP shunt.
• Resection may be effective for VP shunt-related skin metastases.

## 1 Introduction

Ventriculoperitoneal (VP) shunt is a device that is implanted subcutaneously into the abdominal cavity from the lateral ventricle to drain cerebrospinal fluid and is primarily used in the treatment of hydrocephalus. It has been reported that brain tumors can sometimes metastasize into the abdominal cavity via VP shunt, riding the drainage flow from the lateral ventricle. Very rarely, malignant tumors of intra-abdominal organs have been reported to result in abdominal wall metastases along the VP shunt. It is not uncommon for patients with VP shunts to develop colorectal cancer, and several cases of colorectal cancer surgery have been reported. However, to our knowledge, there are no reported cases of VP shunt-related subcutaneous tissue metastasis of colorectal cancer.

We herein report a case of solitary metastatic ascending colon cancer that metastasized along the VP shunt after the primary tumor resection.

This study has been presented in line with SCARE criteria [1].

## 2 Presentation of case

A 79-year-old man was referred to our hospital after a positive fecal occult blood test. He had developed a subarachnoid hemorrhage two years ago and later developed hydrocephalus and had a VP shunt implanted.

**Fig. 1:**
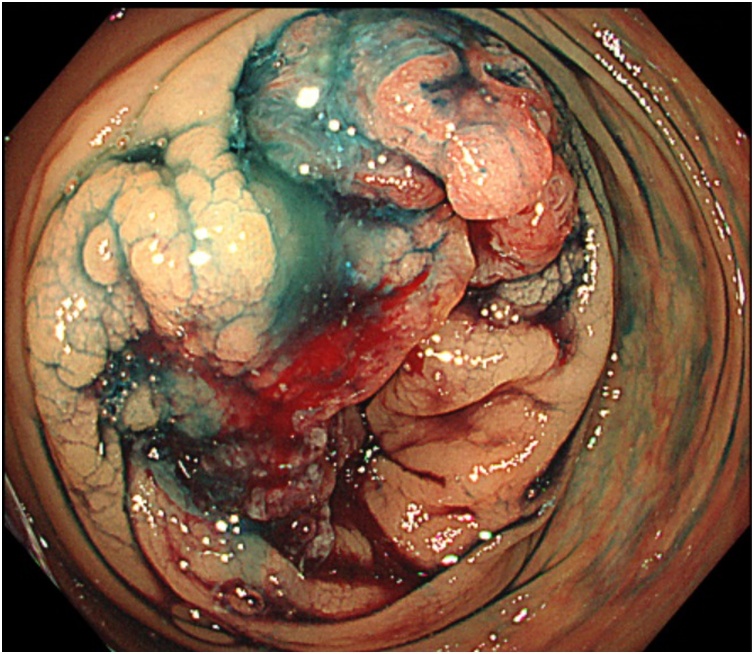
The tumor in the ascending colon at colonoscopy.

A total colonoscopy showed a tumor in the ascending colon (Fig. 1), and the endoscopic biopsy result showed adenocarcinoma. CT scan showed a thickened wall in the ascending colon, but no lymphadenopathy and no distant metastasis were found. We performed laparoscopic right colectomy with mesocolic resction for ascending colon cancer, taking care not to damage the VP shunt intraoperatively. There was no intraoperative dissemination or ascites, no signs of peritoneal carcinomatosis, and no residual tumor. The histopathological diagnosis of the resected ascending colon tumor was well to moderately tubular adenocarcinoma, T4a, N0, M0, and Stage IIB according to the Union for International Cancer Control (UICC) 8th TNM Classification. We followed the patient regularly after the surgery.

**Fig. 2:**
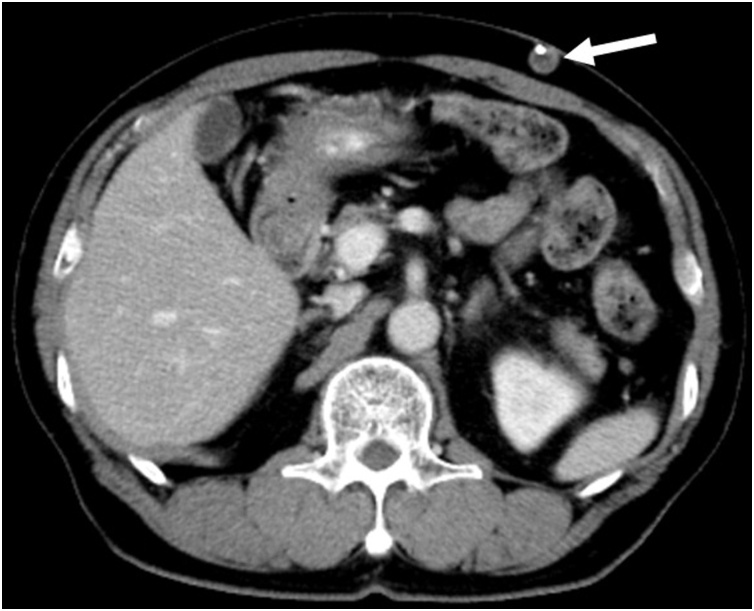
Computed tomography shows a mass in the subcutaneous tissue of the abdominal wall along the VP shunt.

**Fig. 3:**
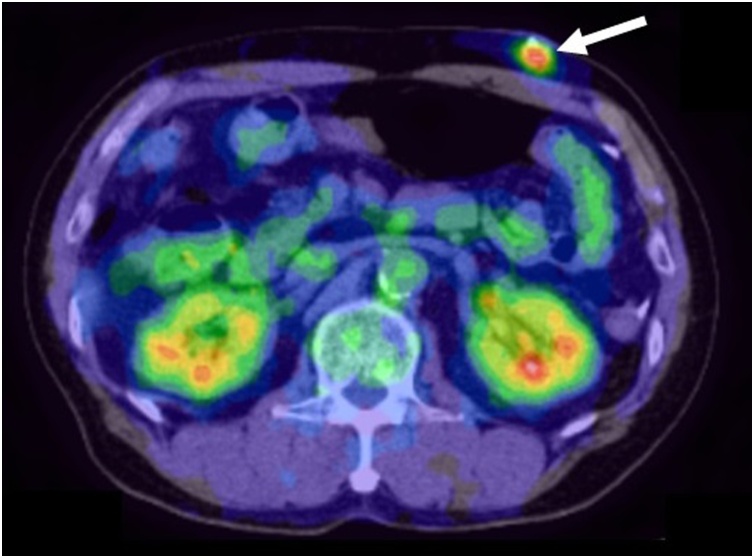
FDG-PET/CT scan showed FDG accumulation on the mass.

At the 6-month post-operative follow-up examination, the patient’s carcinoembryonic antigen (CEA) level was elevated from 3.5 to 42.7. Computed tomography (CT) showed the formation of a mass in the subcutaneous tissue of the abdominal wall along the VP shunt, which was implanted on the left side of the abdomen (Fig. 2). Furthermore, ^18^F-fluorodeoxyglucose positron-emission tomography/computed tomography (FDG-PET/CT) scan showed FDG accumulation coincident with the mass (Fig. 3), suggesting metastasis of colon cancer.

Since it was a single metastasis without other distant metastasis, we performed a resection of the abdominal wall tumor. A spindle-shaped incision was made in the skin directly above the tumor and the subcutaneous tissue was incised with a margin. The catheter of the VP shunt was disconnected from the connection and removed with the tumor. A new catheter was placed into the abdominal cavity by a different route and connected by neurosurgeons. The operative time was 85 min and the intraoperative blood loss was 18 mL.

**Fig. 4:**
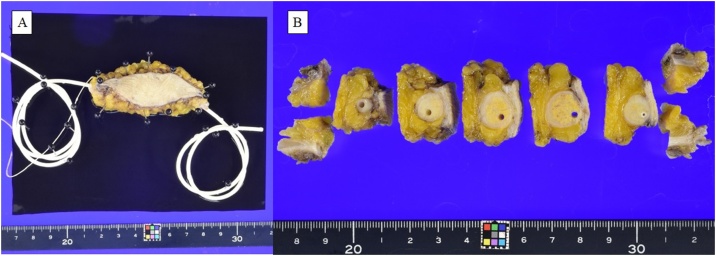
A: Macroscopic inspection of the abdominal wall mass lesion with VP shunt B: Cut surface of the mass.

**Fig. 5:**
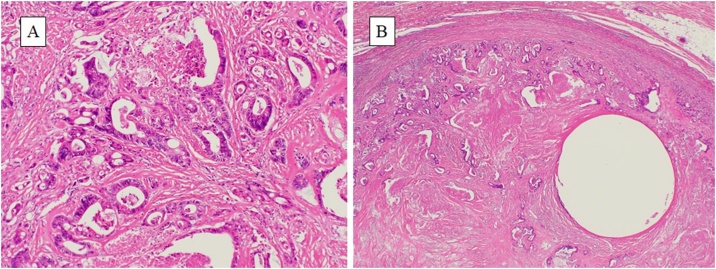
A: The tumor was metastatic ascending colon cancer (hematoxylin and eosin [H&E]; ×100) B: The tumor was present surrounding the VP shunt. (H&E; ×20).

The resection specimen contained a white, 13 mm nodular tumor surrounding the VP shunt (Fig. 4). The histopathological examination confirmed the mass was metastatic ascending colon cancer, with negative margins (Fig. 5). There were no complications during the postoperative course and the patient was discharged on the eighth postoperative day. Postoperative adjuvant chemotherapy was discussed with the patient, but he did not prefer it, so it was not performed. We currently continue his follow up.

## 3 Discussion

Implantation of a VP shunt is a commonly performed procedure for ventricular drainage of hydrocephalus. Although there are some reports of tumor metastases related to the VP shunt in the literature, due to the nature of the drainage of cerebrospinal fluid from the ventricles to the abdominal cavity, most reports are of intraperitoneal metastases of brain tumors [[2], [3], [4]].

Reports of VP shunt-related metastasis due to malignancy of intra-abdominal organs are extremely rare. There are only four cases reported in the literature, which are metastases of pancreatic carcinoma [5], ovarian carcinoma [6], gastrointestinal adenocarcinoma (suspected upper gastrointestinal origin) [7], and gallbladder adenocarcinoma [8], respectively (Table 1). Abdominal wall lesions were present in three of the cases, and only one case of ovarian carcinoma had intraventricular metastasis. Cases of gastrointestinal adenocarcinoma and gallbladder adenocarcinoma were reported as multiple masses along the VP shunt. All of them were highly advanced cases, and metastasis to the VP shunt could not be resected radically. The prognosis was reported to be poor in all cases.

**Table 1:** Summary of reported cases of metastasis of intra-abdominal malignancy along the VP shunt.

Publication year	Author	Sex	Age	Cancer type	Clinical findings
2002	Nawashiro, et al.	Female	61	Pancreatic carcinoma	Single subcutaneous nodule along the VP shunt catheter on the chest.
2008	Eralp, et al.	Female	36	Ovarian carcinoma	Leptomeningeal dissemination in the lateral ventricles of ovarian cancer via a VP shunt. No lesion on the VP shunt catheter.
2017	Halder, et al.	Male	85	Gastrointestinal adenocarcinoma (presumably from the upper gastrointestinal tract)	Multiple subcutaneous nodules along the VP shunt catheter on the chest and abdomen.
2018	Takatu, et al.	Female	75	Gallbladder adenocarcinoma	Several subcutaneous nodules along VP shunt catheter on the scalp, neck, and abdomen.

Abbreviation: VP, Ventriculoperitoneal.

To our knowledge, this is the first case of VP shunt-related subcutaneous tissue metastasis of colorectal cancer to be reported. It is also the first case of an intraperitoneal primary malignancy metastasizing to a VP shunt that was radically resectable.

This case was a metastasis after laparoscopic colon resection, and since the primary tumor had invaded the serosa, it is highly likely that the malignant cells were dispersed in the pneumoperitoneum during the laparoscopic surgery and deposited around the shunt. Another possibility is the presence of tumor cells in the small amount of ascites fluid, which may have attached to the VP shunt and grown. Neale et al. reported that if the pneumoperitoneum pressure did not exceed 80 mmHg, no cerebrospinal fluid reflux occurs in the VP shunt [9]. Therefore, laparoscopic surgery with pneumoperitoneum pressure of 8–10 mmHg generally does not cause reflux in the VP shunt, and there have been many reports of laparoscopic procedures performed with the VP shunt that ended without trouble [10,11].

In this case, cytological examination of cerebrospinal fluid was also performed, but no malignant cells were detected. The fact that the tumor did not grow in the lumen of the VP shunt catheter, but on the outside, suggests that the tumor did not enter the cerebrospinal fluid but rather adhered to the outside of the catheter. It is thought to be a form of metastasis similar to port site metastasis in that it is caused by pneumoperitoneum etc. and metastasizes to the subcutaneous tissue of the abdominal wall. The rate of port site metastasis in colorectal cancer surgery has been reported to be around 1%, and studies have shown that there is no significant difference in wound metastasis of open surgery [12]. As for port site metastasis, the methods for its prevention are still controversial and there is nothing definitive, but it is widely suggested that the surgeons should pay close attention to the surgical operation, such as minimal manipulation of the tumor, avoidance of tumor damage, resection of the tumor with adequate margins, use of protective bags for tissue retrieval, protection of the extraction site, and avoidance of CO_2_ leakage [12,13]. Although these are preventive measures for port site metastasis, we think that similar measures should be taken for cases such as this one.

In the literature, all cases of VP shunt-related metastases are highly advanced and have had a poor prognosis, but we think that they could be cured if they are resected without remnants. Although the incidence is rare, palpation or imaging confirmation of the appearance of nodules in the VP shunt after laparoscopic surgery may lead to early detection of such metastases.

## 4 Conclusion

Although metastasis to the VP shunt after colorectal cancer surgery is rare, it is possible, as in this case. A possible cause of metastasis is that tumor cells exposed on the serosa may have migrated to the VP shunt by pneumoperitoneum. To prevent the development of such metastases, careful intraoperative manipulation is considered essential, and careful postoperative follow-up should be performed.

## Conflicts of interest

None of the contributing authors have any conflicts of interest.

## Sources of funding

All authors have no funding of research.

## Ethical approval

This study is exempt from ethical approval in our institution.

## Consent

Written informed consent was obtained from the patient for publication of this case report and accompanying images. A copy of the written consent is available for review by the Editor-in-Chief of this journal on request.

## Author’s contribution

Masahiro Kataoka: Writing original graft.

Hiroka Kondo: Reviewing.

Yasumitsu Hirano: Supervision, review and editing.

## Registration of research studies

Not applicable.

## Guarantor

Masahiro Kataoka.

## Provenance and peer review

Not commissioned, externally peer-reviewed.
